# Glucose-6-Phosphate 1-Epimerase Responds to Phosphate Starvation by Regulating Carbohydrate Homeostasis in Rice and *Arabidopsis*

**DOI:** 10.3390/plants14243869

**Published:** 2025-12-18

**Authors:** Hongkai Zhang, Shuhao Zhang, Youming Guo, Luyao You, Hongqian Ma, Yubao Cao, Haiying Zhang, Bowen Luo, Xiao Zhang, Dan Liu, Ling Wu, Duojiang Gao, Shiqiang Gao, Baolin Han, Guohua Zhang, Jijin Li, Zihao Feng, Dong Li, Yi Ma, Haibo Lan, Lijuan Gong, Shibin Gao

**Affiliations:** 1 School of Food and Liquor Engineering, Sichuan University of Science and Engineering, Yibin 644000, China; hongkayzhang@126.com (H.Z.);; 2 Sichuan Province Engineering Technology Research Center of Liquor-Making Grains, Yibin 644000, China; 3 Maize Research Institute, Sichuan Agricultural University, Chengdu 611130, China; 4 Key Laboratory of Biology and Genetic Improvement of Maize in Southwest Region, Ministry of Agriculture, Chengdu 611130, China

**Keywords:** low-phosphorus stress, G6PE, carbohydrate homeostasis, *Oryza sativa*, *Arabidopsis thaliana*

## Abstract

Plants adapt to phosphate starvation by remodeling root architecture and reallocating carbohydrates. Glucose-6-phosphate 1-epimerase (G6PE), a key enzyme in carbon and energy metabolism, is hypothesized to contribute to phosphate starvation responses. Here, we investigated the role of G6PE in rice and *Arabidopsis* through phenotypic, physiological, and molecular analyses of *osg6pe* and *atg6pe* mutants. Under normal-phosphate (NP) conditions, both mutants exhibited significantly reduced biomass and fresh weight compared with the wild-type (WT) plants, indicating growth inhibition caused by the mutations. Under low-phosphate (LP) conditions, the mutants displayed enhanced root growth, suggesting that G6PE functions as a negative regulator of radial root growth under phosphate deficiency. The *osg6pe* mutant showed elevated phosphate content and increased leaf starch accumulation under LP, whereas it accumulated more phosphate but less starch under NP. Expression analysis revealed that G6PE transcripts were suppressed under NP but remained relatively stable under LP. Notably, among phosphate starvation-induced (PSI) genes, only *PHT1;4* showed notable transcriptional changes in both species. These findings indicate that G6PE contributes to phosphate homeostasis by modulating carbohydrate metabolism, restraining radial root growth, and selectively regulating *PHT1* expression under phosphate-deficient conditions.

## 1. Introduction

Rice (*Oryza sativa* L.) is one of the world’s most important staple crops, providing dietary energy for more than half of the global population. Phosphorus is an essential macronutrient involved in numerous physiological processes, including energy transfer, nucleic acid metabolism, and photosynthesis [1,2]. The judicious application of phosphorus fertilizer can boost rice yield and improve grain quality [3,4]. However, phosphate rock is a non-renewable mineral resource, and excessive fertilizer use may lead to phosphorus loss and environmental pollution [5]. Due to the rapid fixation or precipitation of phosphorus into insoluble compounds with iron, aluminum, or calcium, a large portion of applied phosphate fertilizers becomes unavailable to plants in agricultural soils [6]. Therefore, understanding the tolerance mechanisms of rice under phosphorus stress provides a theoretical foundation for developing varieties adapted to phosphorus-deficient environments.

Phosphorus is indispensable for the biosynthesis of photosynthetic pigments and the modulation of photosynthetic efficiency. Phosphorus deficiency may hinder the biosynthesis of chlorophyll and other vital pigments, thereby diminishing the efficiency of light harvesting and energy transduction [7]. Photosynthetic rate is largely determined by the rates of ribulose 1,5-bisphosphate (RuBP) carboxylation and RuBP regeneration in the photosynthetic machinery [8]. Phosphorus deficiency reduces stomatal and mesophyll conductance, suppresses rubisco activity, impairs RuBP regeneration, and limits adenosine triphosphate (ATP) production, collectively constraining photosynthetic efficiency [7,9,10]. Under phosphorus-deficient conditions, the accumulation of soluble sugars and starch in leaves promotes carbon partitioning to the roots, thereby enhancing phosphorus acquisition from the rhizosphere [11,12]. The expression of phosphate transporter (*PHT*) genes is modulated by sugar and starch metabolism under phosphate-deficient conditions, facilitating plant adaptation to phosphate-deficient environments [13,14].

Plants have evolved sophisticated adaptive mechanisms to overcome phosphorus deficiency, which is prevalent in natural ecosystems. A critical strategy is the remodeling of root architecture by promoting lateral root elongation and densification, thereby enhancing soil exploration and phosphorus acquisition under phosphorus-deficient conditions [15,16,17]. The secretion of organic acids and phosphatases further facilitates phosphorus acquisition and utilization [18,19]. Additionally, symbiotic relationships with microbes extend the effective root uptake zone, allowing plants to uptake more phosphorus [20,21]. Accumulating evidence supports the idea that the phosphate starvation response (PHR) functions as a central regulator of phosphate starvation signaling. The transcriptional activity of PHR1 is negatively modulated by proteins containing the SIG1/PHO81/XPR1 (SPX) domain, which are encoded by the SPX1-4 genes [22,23]. PHR1 induces the expression of *miR399* and *miR827*, thereby regulating the target genes nitrogen limitation adaption (*NLA*) and phosphate2 (*PHO2*) in response to varying phosphorus conditions [24,25]. Under phosphorus-deficient conditions, PHR1 upregulates PHT1 and phosphate transporter facilitator 1 (PHF1), enhancing transmembrane transport of phosphorus [26].

G6PE is an enzyme that catalyzes the reversible interconversion between α- and β-anomers of glucose-6-phosphate (G6P) [27]. This enzyme participates in the regulation of glycolytic flux and starch biosynthesis, thereby influencing carbon allocation between source and sink tissues. The G6P transporter 1 (GPT1) mediates the transport of G6P into plastids, providing substrates for starch biosynthesis and the oxidative pentose phosphate pathway, processes that are essential for maintaining cellular redox balance and supporting normal plant growth [28]. Previous studies have shown that the maize G6PE homolog *ZmG6PE* regulates carbohydrate homeostasis by modulating the expression of *ZmSPX2* and *ZmPHT1;13*, thereby contributing to phosphate signaling [13]. Despite these insights, the biological functions of plant G6PE remain largely unexplored, particularly regarding its contribution to carbon allocation, sugar signaling, and adaptive responses under nutrient-limited conditions. In this study, we examined the functional roles of the G6PE homologs *OsG6PE* in rice and *AtG6PE* in *Arabidopsis* under LP conditions. Comparative analysis of *OsG6PE*, *AtG6PE*, and *ZmG6PE* may provide cross-species insights into G6PE-mediated responses and deepen our understanding of plant adaptation to phosphorus deficiency.

## 2. Results

### 2.1. Subcellular Localization of OsG6PE and AtG6PE in the Nucleus and Plasma Membrane

**Figure 1:**
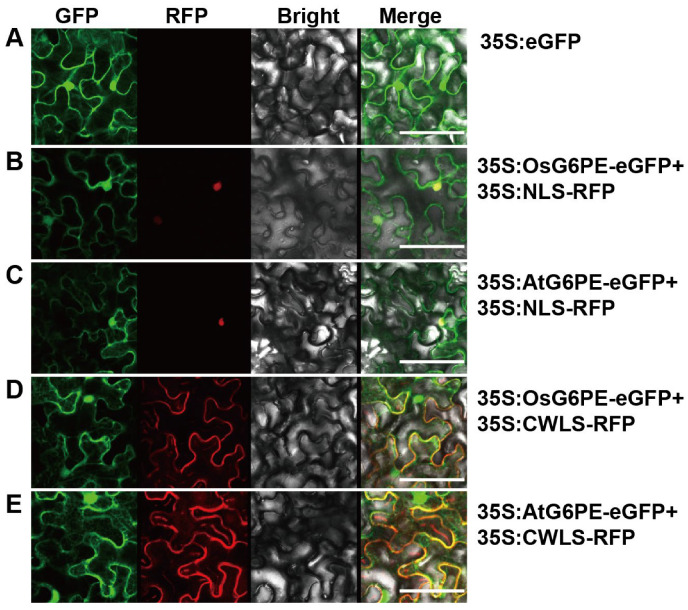
Subcellular localization of OsG6PE and AtG6PE in *Nicotiana benthamiana* leaf epidermal cells. (**A**) Free eGFP expressed from the 35S promoter (35S:eGFP) showing diffuse cytosolic/cortical distribution. (**B**,**C**) OsG6PE–eGFP (**B**) and AtG6PE–eGFP (**C**) co-expressed with the nuclear marker NLS–RFP (35S:NLS-RFP). (**D**,**E**) OsG6PE–eGFP (**D**) and AtG6PE–eGFP (**E**) co-expressed with the cell membrane marker CWLS–RFP (35S:CMLS-RFP). Scale bars = 10 μm.

G6PE possesses broad substrate specificity and contributes to multiple physiological processes. To assess the subcellular localization of *OsG6PE* and *AtG6PE*, the fusion proteins were transiently co-expressed with marker proteins defining distinct organelles. The empty vector control showed only free eGFP (35S:eGFP) signals throughout the cytoplasm and nucleus (Figure 1A). Fluorescence microscopy revealed that both the 35S:OsG6PE and 35S:AtG6PE fusion proteins co-localized with the nuclear localization signal (NLS) marker (Figure 1B,C). Moreover, both fusion proteins co-localized with the cell membrane localization signal (CMLS) marker (Figure 1D,E). These results indicate that OsG6PE and AtG6PE are targeted to both subcellular compartments.

### 2.2. Generation and Characterization of osg6pe and atg6pe Mutants

**Figure 2:**
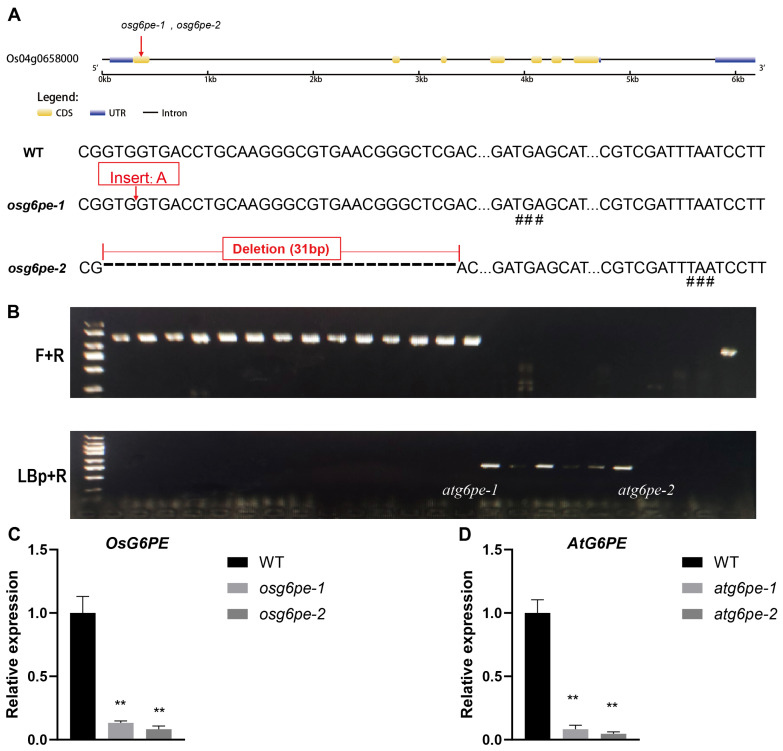
Identification and analysis of mutation sites in *OsG6PE* and *AtG6PE* genes. (**A**) Analysis of the mutation sites in rice mutants *osg6pe-1* and *osg6pe-2*. (**B**) Identification of six homozygous T-DNA insertion mutants in *Arabidopsis thaliana*. (**C**) Expression levels of *OsG6PE* in mutants *osg6pe-1* and *osg6pe-2*. (**D**) Expression levels of *AtG6PE* in mutants *atg6pe-1* and *atg6pe-2*. # indicates a premature stop codon caused by a mutation. Values represent means ± SD (n = 3). Double asterisks (**) indicate significant differences (*p* < 0.01, Student’s *t*-test).

To investigate the function of *OsG6PE*, CRISPR/Cas9-mediated genome editing was employed to generate knockout lines. *osg6pe-1* contains a single-nucleotide deletion, while *osg6pe-2* harbors a 31 bp insertion, both leading to frameshifts and premature stop codons (Figure 2A). Six homozygous T-DNA insertion mutants in *Arabidopsis thaliana* were identified through PCR-based screening of T-DNA insertion sites (Figure 2B). OsG6PE transcript levels were reduced to 13% ± 2% and 8% ± 3% of WT in *osg6pe-1* and *osg6pe-2*, indicating strong knockdown alleles (Figure 2C). AtG6PE transcript levels were reduced to 8% ± 3% and 5% ± 2% of WT in *atg6pe-1* and *atg6pe-2*, representing strong loss-of-function alleles (Figure 2D).

### 2.3. Growth Inhibition of osg6pe and atg6pe Mutants Under NP

**Figure 3:**
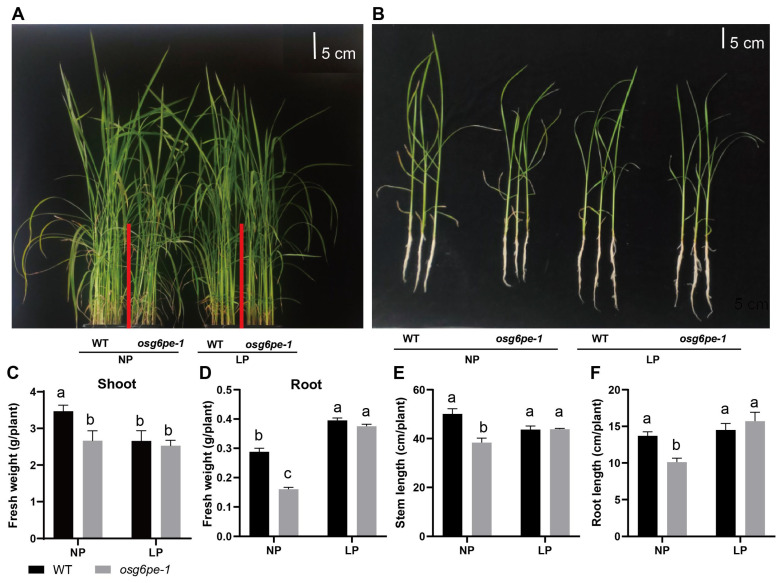
Morphology and growth parameters of rice WT and *osg6pe-1* plants under different phosphate conditions. (**A**) Representative in situ images of WT and *osg6pe-1* growing in hydroponic boxes under NP and LP conditions. (**B**) Representative harvested whole-plant and root system images of WT and *osg6pe-1* after hydroponic culture under NP and LP. (**C**–**F**) Quantitative measurements for leaf fresh weight (**C**), root fresh weight (**D**), shoot length (**E**), and root length (**F**) in WT and *osg6pe-1*. Values represent means ± SD (n = 3). Different letters indicate significant differences among treatments (*p* < 0.05, Waller-Duncan test).

To evaluate the growth performance of *osg6pe* mutants, WT and mutant plants were hydroponically cultured under NP and LP conditions and monitored in situ. Under NP, WT plants exhibited greater overall vigor than *osg6pe-1*, as reflected by taller shoots and more robust aerial biomass (Figure 3A). By contrast, no significant differences in plant size were observed between WT and *osg6pe-1* under LP. Similar trends were confirmed from harvested plants observed ex situ, which also revealed a shorter primary root system in *osg6pe-1* compared with WT under NP, whereas no significant difference was detected under LP (Figure 3B). Consistent results were obtained for the independent rice allele *osg6pe-2* (Supplementary Figure S1A,B) and for *Arabidopsis atg6pe* mutants (Supplementary Figure S2A).

To quantify these phenotypic differences, shoot and root fresh weight, culm length, and total root length were measured after harvest. Under NP, all four traits were significantly lower in *osg6pe-1* than in WT (Figure 3C–F). The independent rice allele *osg6pe-2* showed similar reductions in these growth parameters (Supplementary Figure S1C–F). Likewise, in *Arabidopsis*, both total fresh weight and primary root length were significantly reduced in *atg6pe* compared with WT under NP (Supplementary Figure S2B,C).

### 2.4. OsG6PE Is Involved in Modulating Root Architecture Under Phosphate Stress

**Figure 4:**
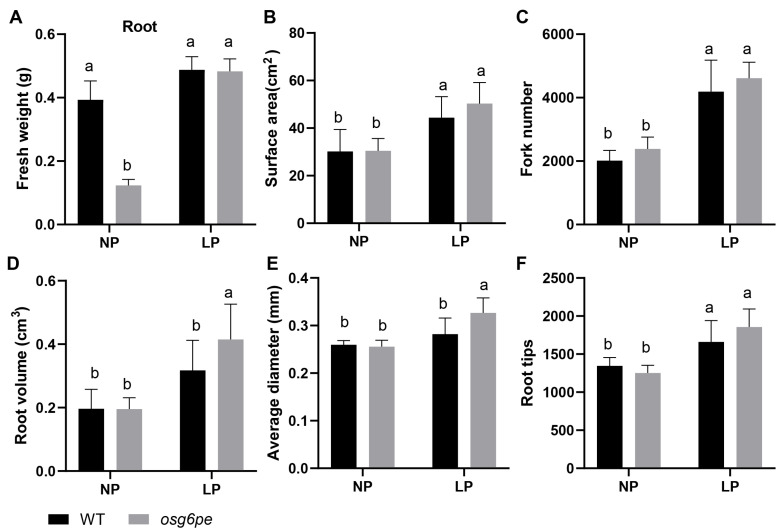
Root system architecture parameters of WT and *osg6pe* mutants under different phosphate conditions. (**A**) Root fresh weight. (**B**–**F**) Quantitative analysis of root traits, including (**B**) surface area, (**C**) number of forks, (**D**) root volume, (**E**) average root diameter, and (**F**) number of root tips. Root traits were measured using WinRHIZO software (version 2016a, 32-bit; Regent Instruments Inc., Québec, QC, Canada). Values represent means ± SD (n = 3). Different letters indicate significant differences among treatments (*p* < 0.05, Waller-Duncan test).

Phenotypic analysis in both rice and *Arabidopsis* revealed that mutations in *OsG6PE* and *AtG6PE* affect root development. Under NP, root fresh weight of the *osg6pe* mutant was significantly lower than that of WT, whereas no significant difference was observed under LP, suggesting that root growth inhibition in the mutant is alleviated under phosphate-deficient conditions (Figure 4A). To quantitatively validate this observation, WinRHIZO root scanning analysis was conducted to assess multiple root architectural traits (Supplementary Figure S3). No significant differences were detected between *osg6pe* and WT in root surface area, fork number, and root tip number, indicating that the mutation has little impact on these architectural parameters (Figure 4B,C,F). Notably, under LP, *osg6pe* exhibited a significantly larger root volume (Figure 4D) and thicker average root diameter (Figure 4E) compared with WT. These observations suggest that OsG6PE serves as a negative regulator of radial root growth under LP conditions, suppressing the radial expansion that leads to increased root volume and thickness in the mutant.

### 2.5. OsG6PE Regulates Carbohydrate Metabolism in Response to LP Stress

**Figure 5:**
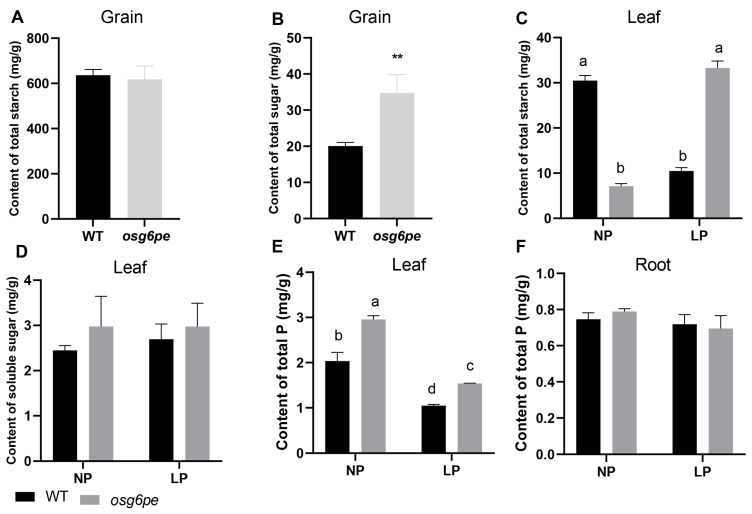
Measurement of starch and soluble sugar contents in rice. (**A**) Starch content in grains. (**B**) Soluble sugar content in grains. (**C**) Measurement of starch content in leaves under different phosphorus conditions. (**D**) Measurement of soluble sugar content in leaves under different phosphorus conditions. (**E**) Leaf phosphorus content. (**F**) Root phosphorus content. Values represent means ± SD (n = 3). Double asterisks (**) indicate significant differences between two groups (*p* < 0.01, Student’s *t*-test). Different letters indicate significant differences among treatments (*p* < 0.05, Waller-Duncan test).

To assess the role of *OsG6PE* in carbohydrate metabolism under phosphate stress, starch and soluble sugar contents were measured in both grains and leaves of *osg6pe* mutants and WT plants. In grains, starch levels did not differ significantly between the mutant and WT (Figure 5A), whereas soluble sugar content was markedly elevated in the *osg6pe* mutant (Figure 5B). In leaves, carbohydrate accumulation exhibited condition-dependent patterns. Under NP, the *osg6pe* mutant exhibited significantly lower starch content compared to WT, while starch accumulation was significantly increased in the mutant relative to WT under LP (Figure 5C). Although soluble sugar levels in mutant leaves tended to be higher than those in WT across both conditions, these differences were not statistically significant (Figure 5D). Notably, leaf phosphorus content in the *osg6pe* mutant was significantly higher than in WT, regardless of phosphate treatment, while root phosphorus levels remained comparable (Figure 5E,F). These results suggest that *OsG6PE* influences carbohydrate partitioning and phosphorus homeostasis in rice, particularly under LP, supporting its role in metabolic adaptation to phosphorus deficiency.

### 2.6. NP Conditions Suppress the Expression of OsG6PE and AtG6PE Genes in the Mutants

**Figure 6:**
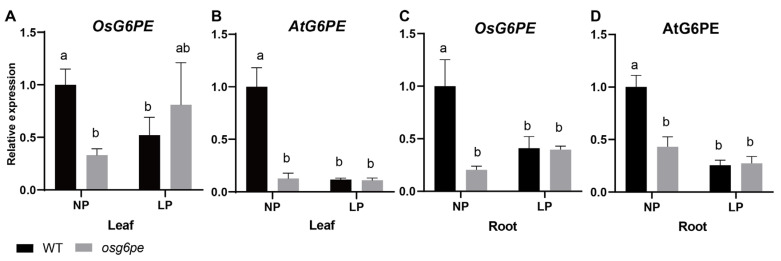
Expression levels of *G6PE* under different phosphorus concentrations. (**A**,**B**) Relative expression levels of *OsG6PE* and *AtG6PE* in leaf tissues under different phosphate conditions. (**C**,**D**) Relative expression levels of *OsG6PE* and *AtG6PE* in root tissues under NP and LP conditions. Values represent means ± SD (n = 3). Different letters indicate significant differences among treatments (*p* < 0.05, Waller-Duncan test).

Under NP, *OsG6PE* expression was significantly lower in the *osg6pe* mutant than in WT, whereas no significant difference was observed under LP stress (Figure 6A,C). A similar pattern was found in *Arabidopsis*, where *AtG6PE* transcript levels were markedly reduced in the *atg6pe* mutant compared to WT under NP, whereas under LP the WT and mutant showed comparably low expression levels (Figure 6B,D). These results suggest that *G6PE* expression is downregulated under phosphate starvation in both rice and *Arabidopsis*, and that the mutation effect is mainly evident under phosphate-sufficient conditions.

### 2.7. LP Conditions Promote the Expression of PSI Genes

**Figure 7:**
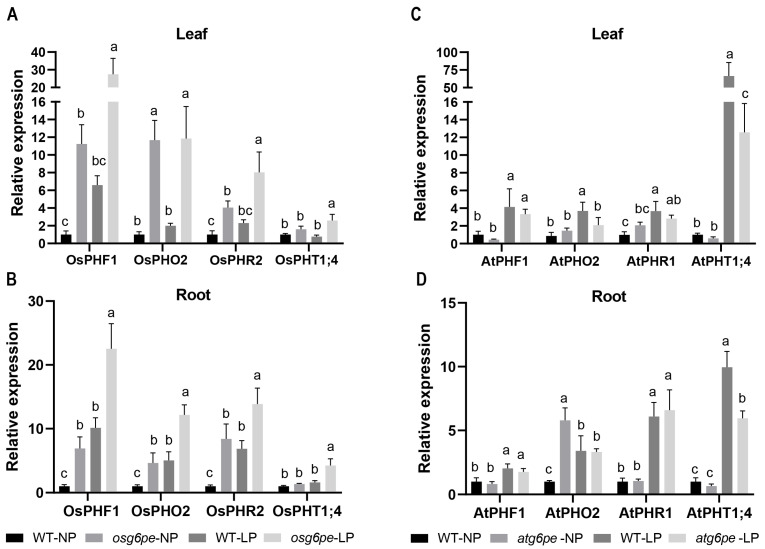
Expression analysis of *PSI*-related genes. (**A**,**B**) Transcript levels of *PSI*-related genes in rice in leaves (**A**) and roots (**B**) grown under different phosphorus conditions. (**C**,**D**) Transcript levels of *PSI*-related genes in *Arabidopsis* in leaves (**C**) and roots (**D**) under different phosphorus conditions. Values represent means ± SD (n = 3). Different letters indicate significant differences among treatments (*p* < 0.05, Waller-Duncan test).

Under NP, the expression levels of *OsPHF1*, *OsPHO2*, and *OsPHR2* were significantly higher in the *osg6pe* mutant compared to the WT (Figure 7A). Similarly, under LP, the expression of *OsPHF1*, *OsPHO2*, *OsPHR2*, and *OsPHT1;4* was also markedly increased in the *osg6pe* mutant relative to WT (Figure 7A). The expression pattern of *PSI* genes in rice roots was similar to that observed in the leaves (Figure 7B). These results indicate that the *OsG6PE* mutation enhances the expression of phosphate-related genes. In *Arabidopsis*, *AtPHO2* exhibits distinct tissue-specific expression patterns. Under LP, the mutant shows a decreased transcript level in leaves (Figure 7C), whereas under NP, its expression is elevated in roots (Figure 7D). Moreover, *AtPHT1;4* displays a consistent expression pattern in both roots and leaves of *Arabidopsis*, with the mutant exhibiting reduced transcript levels under LP (Figure 7D). Notably, expression levels of *OsPHT1;4* and *AtPHT1;4* were significantly altered under LP in both rice and *Arabidopsis*, suggesting a critical role for PHT1;4 in the phosphate starvation response.

## 3. Discussion

### 3.1. G6PE Regulates Carbon Metabolic Balance in Response to Phosphate Starvation

G6P is a key metabolite in glycolysis/gluconeogenesis and the pentose phosphate pathway. G6P has two forms of α and β anomers, and enzymes often exhibit strict specificity toward these anomers [29]. Phosphoglucomutase catalyzes the reversible conversion of α-D-G6P to glucose-1-phosphate, which is subsequently converted by adenosine diphosphate-glucose (ADP-glucose) pyrophosphorylase (AGPase) into ADP-glucose, the primary precursor for starch biosynthesis [30,31]. In contrast, β-D-G6P participates in the pentose phosphate pathway to generate nicotinamide adenine dinucleotide phosphate (NADPH) for cellular metabolism [32,33]. Fructose-6-phosphate and mannose-6-phosphate are also potential substrates of G6PE, indicating its broad substrate specificity [29]. This wide substrate specificity suggests that G6PE may be involved in multiple physiological processes, and that when the enzymatic activities acting on G6P, fructose-6-phosphate, or mannose-6-phosphate are impaired, G6PE may function through a compensatory mechanism to sustain essential metabolic activities. Phosphate starvation stress inhibits photosynthesis and promotes the accumulation of starch and sugars [2,34]. Reduced starch accumulation in the mutant caused a compensatory increase in sucrose levels, thereby modulating phosphate signaling and its crosstalk with nitrogen signaling in rice [14]. Changes in sucrose accumulation and partitioning can modulate phosphate signaling and tissue-specific phosphate-starvation responses [35]. Those studies have demonstrated that phosphate deficiency promotes the accumulation of soluble sugars. Our results demonstrated that LP conditions promoted starch accumulation, consistent with findings from studies on the homologous gene in maize, which may be attributed to the broad substrate specificity of G6PE [13]. Under phosphate-deficient conditions, the soluble sugar content remained stable in rice but declined significantly in maize, implying that G6PE may exhibit distinct regulatory mechanisms of carbohydrate metabolism across species [13]. The preferential accumulation of starch rather than soluble sugars observed in the mutant is likely attributable to the function of G6PE. In WT plants, a functional G6PE helps to channel accumulated sugars into downstream metabolites that participate in various physiological and metabolic processes. However, the loss of G6PE function impairs sugar utilization, resulting in the conversion of excess carbohydrates into more stable and storable starch reserves as an adaptive strategy for maintaining carbon balance under metabolic constraints.

### 3.2. Phosphate Deficiency Induced Remodeling of Root System Architecture

Under phosphate-deficient conditions, plants typically undergo a pronounced remodeling of root system architecture (RSA), characterized by the inhibition of primary root elongation together with the enhanced formation of lateral roots and root hairs [36,37]. Phosphate starvation-induced remodeling of RSA arises from the coordinated action of multiple interdependent regulatory layers, encompassing phosphate signaling, hormone-mediated pathways, carbohydrate availability, local and systemic nutrient sensing, and various structural and rhizosphere-mediated processes [37,38,39]. Within these regulatory layers, carbohydrate availability and sugar signaling have emerged as central integrators that orchestrate RSA remodeling in response to phosphate starvation. Under phosphate-deficient conditions, starch levels in both roots and shoots are significantly elevated [40], yet the role of starch in modulating RSA has not been thoroughly examined. Phosphorus deficiency increases sucrose loading into the phloem and translocation to the roots, reinforcing root sink strength and promoting root proliferation [41,42]. Under phosphate-deficient conditions, alterations in carbon metabolism extend beyond carbon supply and sugar signaling to affect cell wall biosynthesis and cell expansion, thereby modulating root growth [43,44]. The sugar-signaling metabolite trehalose-6-phosphate (T6P) coordinates carbon/energy signaling with auxin pathways to regulate lateral root formation [45]. Although T6P modulates RSA by integrating carbon status with hormonal and energy signals, it remains unclear whether G6P contributes to root architectural remodeling under phosphate-deficient conditions via conversion to T6P. Phosphate uptake mediated by high-affinity PHT1 transporters, together with carbon signaling, coordinates root carbon allocation, thereby enhancing lateral root and root hair development and ultimately optimizing RSA under phosphate-deficient conditions [42,46]. The enhanced root volume and diameter in the *osg6pe* mutant under LP may result from the loss of OsG6PE-mediated repression. These findings suggest that OsG6PE functions as a negative regulator of radial root growth, potentially by modulating carbohydrate allocation and the expression of *PHT1* transporters under phosphate-deficient conditions.

### 3.3. The Role of PSI Genes Responded to LP Stress

In both *Arabidopsis* and rice, PHF1 is crucial for regulating the localization of high-affinity inorganic phosphate transporters to the plasma membrane [26]. Overexpression of *OsPHF1* enhances phosphate accumulation in shoots and roots under phosphate-sufficient conditions [47]. Conversely, *atphf1* mutants exhibits a significant reduction in phosphate uptake, resulting in decreased phosphorus content in shoots and roots, as well as impaired growth and development [26]. Our findings indicate that *PHF1* expression exhibits divergent patterns in rice and *Arabidopsis*, consistent with species-specific regulatory mechanisms and phenotypic responses to phosphorus deficiency.

*AtPHO1* is predominantly expressed in roots, and its transcript levels are elevated under phosphorus starvation [48]. *AtPHO2* is expressed in both roots and leaves, implying a potential role in regulating phosphate translocation between these tissues [49]. OsPHO1;1 participates in interaction among phosphate, zinc, and iron, contributing to regulation of iron transport by integrating phosphate- and zinc-deficiency signals [50]. OsPHO1;2 plays a pivotal role in translocation phosphorus from rice roots to aerial tissues, and the *ospho1;2* mutant exhibits impaired grain filling due to the suppression of AGPase activity [51,52]. These studies demonstrate that PHO homologs play distinct roles in responses to phosphate starvation, which is consistent with our expression analysis of *PHO* genes under LP.

In rice, overexpression of *OsPHR2* activates phosphate starvation-responsive genes even under phosphate-sufficient conditions, leading to excessive phosphate accumulation in shoots and inhibition of root growth [53,54]. In *Arabidopsis*, mutation of *AtPHR1* attenuates the root hair elongation response to phosphate deficiency [55,56]. These findings indicate that PHR plays a conserved role in phosphate signaling and may indirectly influence root development by regulating phosphate starvation-responsive genes. In our study, *PHR*-family expression was upregulated under LP, which may contribute to the regulation of root elongation. Under phosphate deficiency, plants exhibit a pronounced redistribution of carbon metabolism to sustain root growth and phosphate starvation responses [43,57]. Given the central role of G6PE in carbon metabolism, the potential interaction between PHR and G6PE merits further investigation.

In higher plants, the PHT1 family comprises numerous members that share some spatiotemporal expression patterns while also exhibiting marked differences. The high transcript levels of *AtPHT1;1* and *AtPHT1;4* in roots are associated with their roles as high-affinity phosphate transporters [58]. OsPHT1;4 is involved in phosphate uptake and translocation, playing a crucial role in rice embryo development, while OsPHT1;8 is key for phosphate allocation and remobilization [59,60]. ZmG6PE is implicated in phosphate uptake through the regulation of *ZmPHT1.13* expression [13]. However, G6PE response pattern under phosphate stress in maize differs from that in rice and *Arabidopsis*, likely due to differential expression of G6PE homologs or *PSI* genes. Evidence shows that G6PE affects carbohydrate homeostasis and thereby modulates *PHT* gene transcription. Consistently, our study demonstrates that OsG6PE negatively regulates radial root growth and influences carbon allocation and *PHT1*;4 expression under phosphate limitation. The enhanced root growth and altered *OsPHT1;4* expression in the *osg6pe* mutant further support a link between carbohydrate metabolism and phosphate transporter regulation.

## 4. Materials and Methods

### 4.1. Plant Materials and Growth Conditions

Using the clustered regularly interspaced short palindromic repeats (CRISPR)/CRISPR-associated protein 9 (Cas9) system, we successfully knocked out the *OsG6PE* gene in the conventional japonica rice cultivar Zhonghua 11 (ZH11), thereby generating *osg6pe* mutants. The *Arabidopsis* T-DNA insertion mutant SAIL_060593.53.50.X was acquired after confirming its identity through the official website (https://www.arabidopsis.org/, accessed on 1 December 2025). The primers used for genotyping *osg6pe* and *atg6pe* mutants are listed in Supplementary Table S1.

Full and healthy rice seeds were selected for germination, followed by hydroponic cultivation in deionized water until the three-leaf stage. Plants were cultured in a modified Hoagland nutrient solution containing 1 mM phosphate for the NP treatment and 1 μM phosphate for the LP treatment. The hydroponic nutrient solution was replaced every three days and aerated for eight hours daily. The *osg6pe-1* and *osg6pe-2* phenotyping experiments were conducted as two independent batches grown in different seasons. Seasonal variation, which led to differences in harvest time, caused variation in the absolute biomass and root length of WT plants between batches. All statistical analyses were performed separately within each batch, and the phenotypic trends associated with each genotype remained consistent across experiments.

*Arabidopsis* seeds were surface-disinfected with 75% ethanol on a super-clean bench for two minutes, followed by rinsing with sterilized ddH_2_O to remove any residual disinfectant. The sterilized seeds were sown on half-strength Murashige and Skoog (1/2 MS) agar plates supplemented with different phosphorus concentrations and cultured in an *Arabidopsis* growth chamber.

### 4.2. Reverse Transcription–Quantitative Polymerase Chain Reaction (RT-qPCR) Analysis

The total RNA was extracted using a Trizol reagent kit (Invitrogen, Thermo Fisher Scientific, Waltham, MA, USA; Cat. No. 15596026) according to the manufacturer’s instructions. First-strand cDNA was synthesized from 1 µg of total RNA using the PrimeScript™ RT Reagent Kit with gDNA Eraser (Takara Biomedical Technology, Beijing, China; Cat. No. RR047A) according to the manufacturer’s instructions. Gene-specific primers were designed with Primer3 (https://primer3.ut.ee/, accessed on 1 December 2025), and their sequences are listed in Supplementary Table S1. *OsActin1* (rice) and *AtUBQ10* (*Arabidopsis*) were used as reference genes due to their stable expression under phosphate treatments. RT-qPCR was performed with FastStart Essential DNA Green Master (Roche, Basel, Switzerland; Cat. No. 06402712001) on a Bio-Rad real-time PCR system (Bio-Rad Laboratories, Hercules, CA, USA). Each sample included three biological replicates and three technical replicates. Relative transcript levels were calculated using the 2^−ΔΔCt^ method, and data processing was conducted with Bio-Rad CFX Manager v4.0. The relative expression level of WT was normalized to 1, and the error bars represent the standard deviation of the ΔCt values from biological replicates.

### 4.3. Starch and Soluble Sugar Content Determination

Leaf starch content was quantified using a starch assay kit (Grace Biotechnology, Suzhou, China; Cat. No. G0507W) according to the manufacturer’s instructions. Soluble sugar content was measured using a soluble sugar assay kit (Comin Biotechnology, Suzhou, China; Cat. No. KT-1-Y) following the manufacturer’s protocol.

### 4.4. Determination of Phosphorus Content

Fresh biomass was oven-dried at 105 °C to a constant weight. The dried material was ground to a fine powder, and 0.20 g was accurately weighed and digested in a mixture of H_2_SO_4_ and H_2_O_2_. The digest was diluted to a final volume of 50 mL. Total phosphorus content was determined using a SmartChem 200 discrete autoanalyzer (Unity Scientific, Brookfield, WI, USA).

### 4.5. Subcellular Localization Analysis

The full-length coding sequence was cloned into the pCAMBIA2300-eGFP vector to generate a recombinant plasmid, which was subsequently introduced into *Agrobacterium tumefaciens*. The bacterial culture was harvested by centrifugation and resuspended in 10 mM MgCl_2_ containing 150 µM acetosyringone (Sigma-Aldrich, St. Louis, MO, USA). The resulting suspension was infiltrated into the abaxial surface of *Nicotiana benthamiana* leaves using a needleless syringe. After incubation in the dark for 48 h, fluorescence signals were observed under a confocal laser scanning microscope (Leica TCS SP8; Leica Microsystems, Wetzlar, Germany).

### 4.6. Statistical Analysis

All experiments were performed with three independent biological replicates, and each biological replicate consisted of independently grown plant materials. Statistical significance was assessed using two-tailed Student’s *t*-test for comparisons between two groups, or one-way ANOVA followed by the Waller-Duncan multiple range test for comparisons among more than two groups. A significance level of *p* < 0.05 was applied. Error bars represent standard deviation (SD).

## 5. Conclusions

This study using *osg6pe* and *atg6pe* mutants in rice and *Arabidopsis* revealed that G6PE functions as a negative regulator of phosphate starvation responses. Under LP conditions, the mutants showed increased root volume and thicker roots, accompanied by increased leaf starch accumulation, and exhibited altered expression of phosphate transporter genes such as *PHT1;4*. These results indicate that G6PE coordinates carbon metabolism, root morphological plasticity, and the selective regulation of *PHT1* genes to maintain phosphate homeostasis. The conserved function of G6PE across monocot and dicot species provides a basis for improving crop adaptation to phosphate-deficient environments and enhancing phosphorus use efficiency.

## Data Availability

The original contributions presented in this study are included in the article/Supplementary Material. Further inquiries can be directed to the corresponding author.

## Supplementary Materials

The following supporting information can be downloaded at https://www.mdpi.com/article/10.3390/plants14243869/s1.
