# Supplementary material for: Glucose-6-Phosphate 1-Epimerase Responds to Phosphate Starvation by Regulating Carbohydrate Homeostasis in Rice and Arabidopsis
Source: Plants (Basel). 2025 Dec 18;14(24):3869. doi: 10.3390/plants14243869 (PMC12736901; doi:10.3390/plants14243869)
Supplement: Supplementary file 1 [file plants-14-03869-s001.zip › Supplementary Figures.pdf]

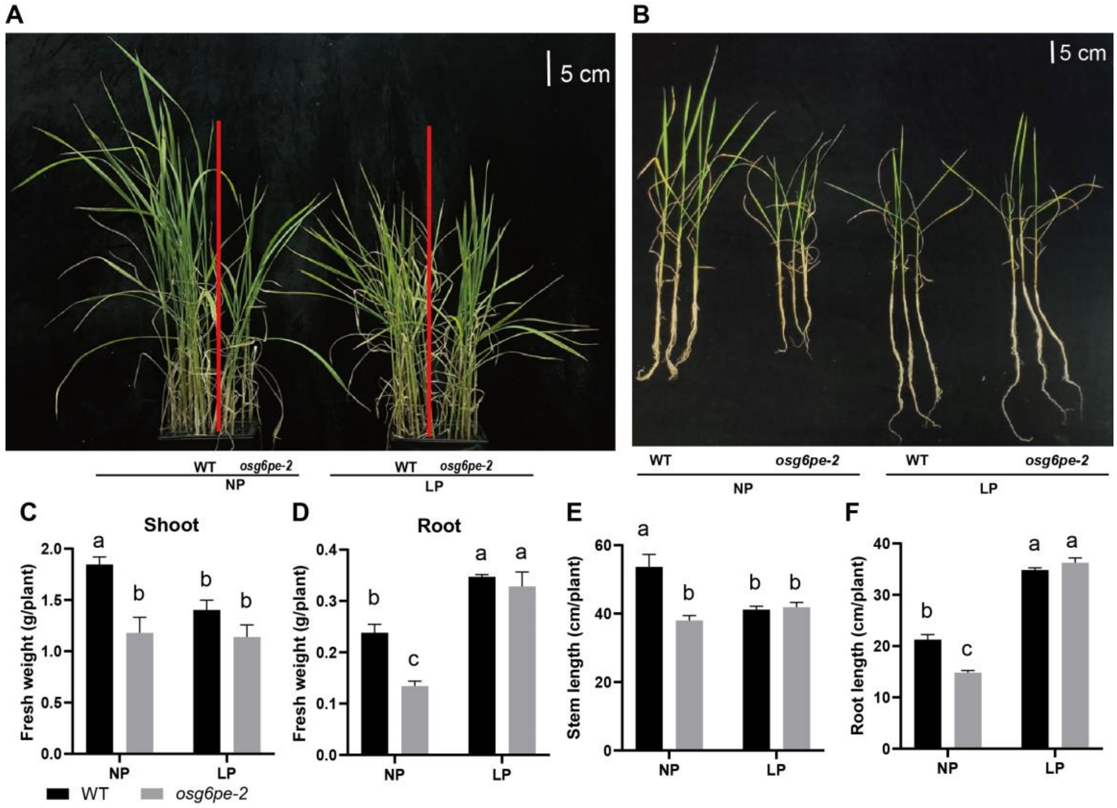

Supplementary Figure S1. Verification of phenotypes in *osg6pe-2* and additional in situ observations.

(A–B) Representative in situ (A) and harvested (B) images of WT and *osg6pe-2* under NP and LP. (C–F) Quantification of leaf fresh weight per plant (C), root fresh weight per plant (D), shoot length (E), and root length (F) of WT and *osg6pe-2*. Values represent means ± SD (n = 3). Different letters indicate significant differences among treatments ($P < 0.05$, Waller-Duncan test).

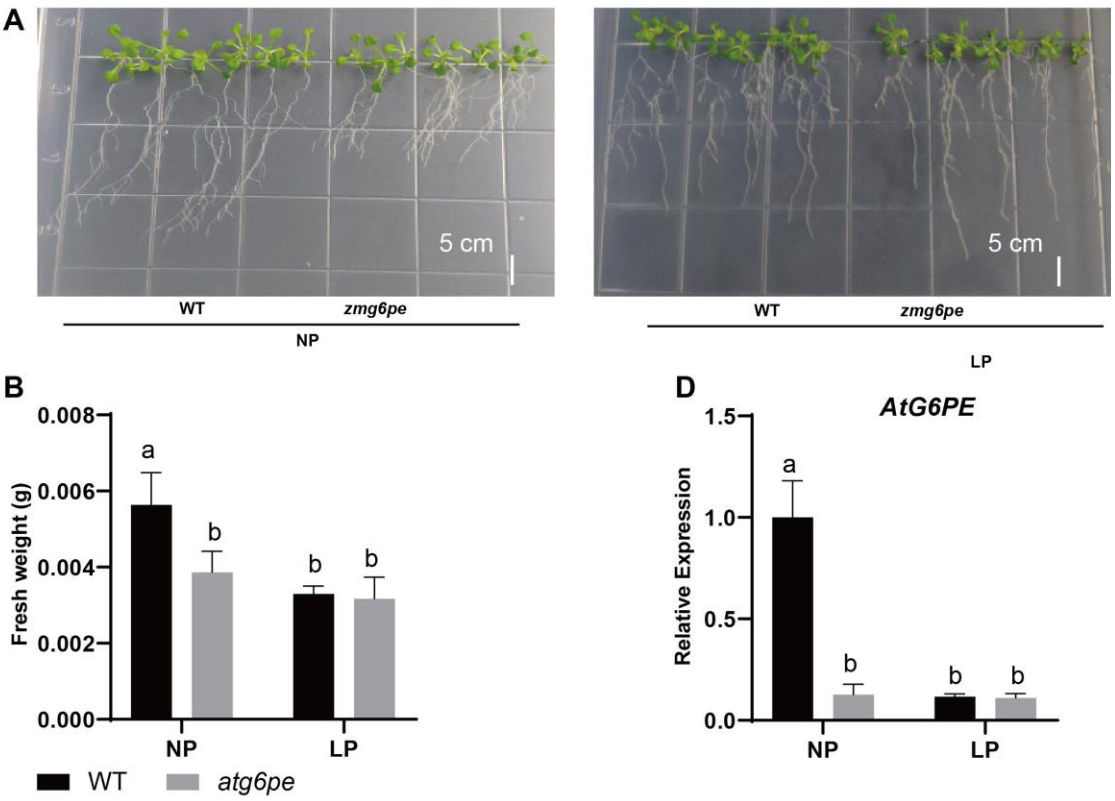

Supplementary Figure S2. Growth phenotypes and quantitative analysis of *Arabidopsis thaliana* WT and *atg6pe* mutant under different phosphate regimes.

(A) Representative images of WT and *atg6pe* seedlings grown on solid medium under NP and LP conditions. (B–C) Quantification of shoot fresh weight per plant (B) and primary root length (C) of WT and *atg6pe* seedlings under NP and LP conditions. Values represent means ± SD (n = 3). Different letters indicate significant differences among treatments (P < 0.05, Waller-Duncan test).

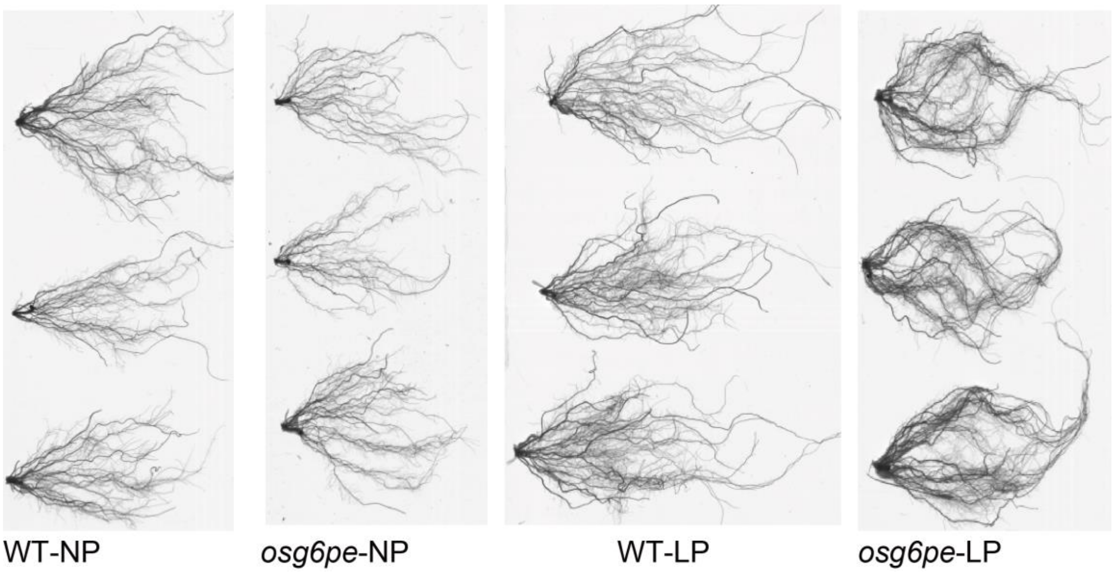

Supplementary Figure S3. Representative scanned images of root systems of WT and *osg6pe* mutants under phosphate treatments.
